# Patient Engagement in Medical Education During the COVID-19 Pandemic: a Critical Reflection on an Epistemic Challenge

**DOI:** 10.15694/mep.2021.000103.1

**Published:** 2021-04-30

**Authors:** Julie Massé, Guy Poulin, Marilyne Côté, Marie-Claude Tremblay

**Affiliations:** 1 Université Laval; 2 VITAM; 3 Mobilisation régionale agir ensemble contre les préjugés

**Keywords:** Epistemic Injustice, Patient Engagement, Patient Involvement, Patient Participation, Experiential Knowledge, Medical Education, Power, Reflexivity, Online Teaching, COVID-19

## Abstract

This article was migrated. The article was not marked as recommended.

Epistemic injustices are defined as inequalities of power in the access, recognition and production of knowledge. Their persistence in medical education, especially to the detriment of patients and their specific knowledge, has been documented by several authors. Patient engagement is a new paradigm that involves fostering meaningful patient collaboration at different levels of the healthcare system. Since it is fundamentally based on the recognition of the value and relevance of patients’ experiential knowledge, patient engagement in medical education is generally recognized as a desirable strategy to address epistemic injustices in the field. Patient engagement is challenged in the context of COVID-19 where most Canadian medical schools have had to modify their teaching models in a short period of time, stop in-person classes and redirect most activities online. This article presents a critical reflection on the issues raised by COVID-constrained teaching strategies and their impact on epistemic injustices in medical education. It also suggests strategies to favour epistemic justice in medical education despite the pandemic turmoil and online shift. It therefore adds an epistemological perspective to the reflection on the effects of the pandemic on medical education and training, which has been little discussed so far.

## Background

The notion of epistemic injustice has emerged under contemporary feminist and postcolonial epistemologies to help rethink the links between social inequalities and knowledge production ( Godrie and Dos Santos, 2017). On this basis, epistemic injustices were defined as inequalities of power in the access, recognition and production of knowledge ( Fricker, 2007; Godrie and Dos Santos, 2017). These injustices fundamentally arise from broader systemic inequalities and situations of oppression structuring and nurturing social relations in various contexts ( Godrie and Dos Santos, 2017). Their persistence in medical education, especially to the detriment of patients and their specific knowledge, has been documented by several authors ( Bleakley, Bligh and Browne, 2011; Sharma, 2018). Notably, it has been argued that traditional social dynamics in medical education, rooted in the long-lasting culture of a health system that has fundamentally built itself on expert knowledge ( Dumez and Pomey, 2019), “led to and perpetuated the types of power imbalances that excluded and alienated patients” ( Bleakley, Bligh and Browne, 2011, p. xiii).

Patient engagement is a new paradigm that involves fostering meaningful patient collaboration at different levels of the healthcare system, including direct care, health service organization, health policies and medical education ( Pomey *et al.,* 2015). It is the culmination of a logical evolution, over several decades, of ways of thinking about health and organizing health care and services ( Dumez and Pomey, 2019). Since it is fundamentally based on the recognition of the value and relevance of patients’ experiential knowledge ( Tuckett *et al.,* 1985; Pomey *et al.,* 2015; Dumez and Pomey, 2019), patient engagement in medical education is generally recognized as a desirable strategy to address epistemic injustices in the field. Indeed, meaningful participation of patients, i.e., going beyond the historically instrumental role granted to patients in medical education, has the potential to enable patients to bring needed experiential knowledge in the training of future physicians and to consequently constitute themselves as holders and creators of valued knowledge.

That said, patient engagement is challenged in the context of COVID-19 where most Canadian medical schools have had to modify their teaching models in a short period of time, stop in-person classes and redirect most activities online to allow social distancing and curb the spread of the virus. In Quebec (Eastern Canadian province), the Premier spoke to the citizens on March 12, 2020, to announce a first wave of public health measures. The following day, the provincial Ministry of Education and Higher Education confirmed the suspension of all classroom training and evaluation activities and the introduction of specific measures applicable to the province’s universities with respect to the maintenance of distance education. Although government guidelines have evolved over time and by regions since then, training activities in Quebec universities remain mostly online at the time of writing this article. The situation is comparable in other Canadian provinces.

In this article, we present a critical reflection on the issues raised by COVID-constrained teaching strategies and their impact on epistemic injustices in medical education. We also suggest strategies to favour epistemic justice in medical education despite the pandemic turmoil and online shift. The article therefore adds an epistemological perspective to the reflection on the effects of the pandemic on medical education and training, which has been little discussed so far.

## Fricker’s theoretical proposition on epistemic injustices

Our critical reflection will be based on the theoretical proposition of Miranda Fricker (2007), a contemporary British philosopher whose work is at the very origin of the notion of epistemic injustice. According to Fricker (2007), there are two kinds of epistemic injustices: testimonial injustice and hermeneutical injustice. The next sections will attempt to describe these two types of epistemic injustices in further detail and present how they actually apply to patient engagement in medical teaching in the pandemic context.

## Patient exclusion in the pandemic context: source of epistemic injustice?

### Patient exclusion as testimonial injustice

Following Fricker (2007), **testimonial injustice** involves a hearer attributing an unjust credibility deficit to a certain speaker due to prejudice, bias and stereotyping. The author depicts this type of epistemic injustice “as a kind of injustice in which someone is wronged specifically in his/her capacity as a knower” (p. 20). Indeed, it implies a mechanism through which identity prejudice distorts the hearer’s judgement of the speaker’s credibility, which results in that speaker’s disqualification as a knower. Ultimately, “this operation of identity power controls who can convey knowledge to whom and, by the same token, who can gain knowledge from whom” (p. 90).

### How does testimonial injustice apply to medical education in the COVID-19 context?

One of the first impacts of the COVID-19 crisis has been that, in the medical field, in light of the emergency measures that had to be taken, decision-makers redesigned activities in a manner that resulted in the exclusion of patients from many participatory platforms. As outlined by other authors ( Drinkwater, 2020; Richards and Scowcroft, 2020), the way patient participation was quickly and easily stopped in various contexts raises fundamental questions about how it is really valued. From an epistemic perspective, such exclusion highlights pervasive power imbalance in which power holders in the medical field still tend to exclude patients as epistemic agents, holding valuable knowledge of their own. In the face of adversity, we have indeed quickly returned to traditional often-criticized paternalistic logics ( Bleakley, Bligh and Browne, 2011; Sharma, 2018) where physicians and academics are the sole holders of authority, credibility and legitimacy to create and share knowledge, and where patients are disempowered and relegated to their status of objects of medical knowledge.The testimonial injustice here lies in the fact that experiential patients’ contributions in the medical field, based on knowledge and skills gained through experiences of those living with the disease and the use of health services, is disqualified (since judged non-essential given the emergency circumstances) in favour solely of biomedical expert knowledge.

In the current pandemic context where most training activities are transferred online, we can also establish a link between testimonial injustices and digital inequalities, whose increase in time of COVID-19 has been demonstrated ( Beaunoyer, Dupéré and Guitton, 2020). In the particular context of medical education, we are aware that some institutions managed to maintain patient engagement alive in medical teaching despite the COVID-constrained educational context (for instance, the Université de Montréal Collaboration and Patient Partnership Unit’s initiative in the context of which patient teaching roles have rapidly moved online) ( Descoteaux and Jackson, 2020). One of the most commonly preferred strategy to do so lied in online meeting platforms (e.g. Zoom, Teams, Webex), which now often replace in-class group interactions that took place before the crisis. Even if we clearly see the practicality of such socially-distanced synchronous teaching approaches in the pandemic context, they still raise unavoidable epistemic issues. As digital technologies become the prominent way to interact, participate and learn in medical education under the impetus of the pandemic, inequalities in access to and ability to use technologies may limit the range and scope of patient engagement. Indeed, digital inequalities make it potentially complex for people or communities who have difficulty accessing or using technologies due to financial, geographic, social or literacy-related barriers, to access participatory spaces and then properly engage in knowledge co-construction. While Beaunoyer *et al.* (2020) highlight how digital inequalities are distributed along a continuum influenced by socio-economic status, age, education, migration status and health literacy, failure to adequately take into account the barriers to online participation faced by some individuals and groups in the design of educational strategies represents the basis for testimonial injustices. Indeed, Fricker (2007) underlines that some significant form of testimonial injustice occurs “when prejudice [...] leads to a tendency for some groups [who are subject to identity prejudice] simply not to be asked for information in the first place” (p. 130). The testimonial injustice thus lies in the fact that the non-reflexive use of digital participatory tools will be silencing the voice of some, who will be unjustly denied the opportunity to share their knowledge in medical education since they are not given the means to do so. Following Fricker (2007),such exclusion lies in the fact that those individuals and social groups still do not constitute *good informants,* with regard to their capacity to produce knowledge that is conceived a priority from the dominant perspective. Engaging populations experiencing circumstances of vulnerability was already recognized as a major challenge before the pandemic ( Loignon *et al.,* 2019; Pomey *et al.,* 2019). The shift to online activities is widening disparity since it may reinforce the current trend toward the recruitment of well-resourced, educated and privileged patients ( Sharma, 2018).

### Patient exclusion as hermeneutical injustice

Fricker (2007) defines **hermeneutical injustice** as “the injustice of having some significant area of one’s social experience obscured from collective understanding owing to hermeneutical [i.e.: interpretative] marginalization” (p. 158). It thus refers to unjust inequalities in the intelligibility of social experiences, given the collectively-accepted interpretative schemes for understanding the world. Following Fricker (2007), the misinterpretation of one’s social experience often results from his/her traditional exclusion from activities meant to shape collective interpretative schemes. In this regard, the author’s work highlights how the experience of some women (relating to sexual harassment, for example) is less intelligible because women have historically been excluded from activities meant to shape the way we collectively interpret the world (e.g.: scholarship, journalism, politics). As Fricker(2007) claims that this is also the case for other marginalized groups, we consider that it is the case of patients as “[the] expert health system [...] has defined itself for decades without its users, patients, and citizens” (Dumez and Pomey, 2019, p.14), and patients have thus traditionally been excluded from the activities meant to shape the way we collectively understand health and disease.

### How does hermeneutical injustice apply to medical education in the COVID-19 context?

Scientific literature ( Domecq *et al.,* 2014; Carroll *et al,* 2017) suggests the main challenges to patient engagement often lie in the perceived logical and practical burden associated with it (e.g., the time and energy required to develop a common language, to redefine the roles and to value patient contribution in a way that avoids tokenism). In the face of the pandemic turmoil, we postulate medical teachers might be inclined to avoid such a burden and seek alternative strategies to somehow maintain students’ exposure to patients’ perspectives in a relatively simple, timely and energy saving way. For instance, medical teachers may be tempted to fall back on the online publication of asynchronous patient testimonials. At first sight, the formative character of such practice appears relevant since it contributes to the contextualization of one person’s case and, in doing so, provides better understanding of the issues raised and fosters the acquisition of the skills required to properly address it ( Lechopier and Granier, 2017); however, it does not restore the patient’s role as co-constructor of knowledge. Indeed, in the absence of meaningful interactive patient-student contacts allowing for authentic dialogue (favouring the development of common interpretive schemes), unilateral patient testimony risks resulting in academic and clinical teachers using the patient’s narrative as a tool to illustrate clinical or biomedical knowledge ( Lechopier and Granier, 2017). In other words, the patient’s narrative acts “as interesting teaching ‘material’, often no more than a medium through which the teacher teaches” ( Spencer *et al.,* 2000, p. 851).

The hermeneutical injustice thus lies in the potential inability for medical teachers - and medical students - to make sense of the patient’s experience because the collectively accepted dominant biomedical paradigm they refer to (and the interpretative resources it implies) does not fit the paradigm within which the patient’s narrative is forged. The tendency will be to understand the disease in terms of vital norms and objective indicators, to reduce it to its physiological dimension, in a logic that is disconnected from the patient’s lived and signified reality ( Vonarx and Desgroseilliers, 2013). The patient’s unique subjective experience thus risks being assimilated to an objectified, dehumanized and universalized ‘patient perspective’ ( Rowland *et al.,* 2017; Sharma, 2018), and to be instrumentalized in the reproduction of existing power dynamics and ideological systems rather than possibly being used to resist or transform them ( Rowland *et al.,* 2017).

## Possible solutions toward a sustainable epistemic justice in medical education

We have highlighted how COVID-constrained teaching strategies can exclude patients from the process of knowledge co-construction in medical education and thus constitute a source of epistemic injustice. Knowing that COVID-19 will likely affect us for a long time and that many institutions now consider virtual teaching practices as the way forward, the issues raised highlight the need to initiate a reflection on appropriate strategies to develop a sustainable renewed model for active and equitable patient engagement in medical education. Consistent with Fricker’s proposal (2007), we consider that educational strategies should be rooted in the principles of testimonial and hermeneutical justice.

From both testimonial and hermeneutical perspectives, solutions toward epistemic justice as proposed by Fricker(2007) mainly rely on the hearers’ development of a reflexive awareness to ultimately change how he perceives and engages with the speaker. In doing so, Fricker’s proposal intersects with some iterative models of reflexivity ( Schön, 1983; Sandars, 2009). From the testimonial perspective, the reduction of epistemic inequalities in medical education may be achieved through solutions “neutraliz[ing] the impact of all sorts of prejudices endemic in the climate of testimonial exchange” ( Fricker, 2007, p. 96). Following Fricker (2007), such neutralization requires the hearer to engage in an active critical reflection to question his spontaneous perception of the speaker’s words, to become aware that this perception is not neutral since it is the result of an identity-based power that mediates between him and the speaker and to strategize to “compensate upwards” (p. 91-92). As for solutions based on hermeneutical justice, they will seek to promote the hearers’ development of a reflexive awareness for the existence of gaps in collective interpretative resources that can be mobilized to understand some speakers’ message ( Fricker, 2007). Indeed, medical teachers and students may reflexively question how their social identity, the very foundations of their interpretative schemes, the biomedical paradigm they were socialized in and the power dynamics it involves, limit their in-depth understanding of the patient’s discourse and potentially make them miss out on fundamental learning.

Following Fricker (2007), in order to support the hearers’ reflexive efforts, educational strategies promoting “a more inclusive hermeneutical micro-climate through the appropriate kind of dialogue” (p. 171) between teachers, students and patients should be prioritized within the medical curriculum. This implies that interventions involving “a more pro-active and more socially aware kind of listening than is usually required in more straightforward communicative exchanges” ( Fricker, 2007, p. 171) should be sustainably implemented and adequately supported. The Université de Montréal’s initiative ( Descoteaux and Jackson, 2020) shows us that the pandemic emergency and the online shift are not necessarily an obstacle to the implementation of such educational strategies; however, in that particular context, attention should be paid to digital inequalities to ensure equitable access to knowledge sharing opportunities. Beaunoyer *et al.*’s (2020) proposition of a set of sustainable action-focused strategies targeting equitable access to and use of technologies may be particularly useful to act in that way. For instance, Patient Service Centres could be implemented to offer: (i) training courses aimed at increasing patients’ digital literacy; (ii) equipment loan services, with geographically and physically accessible service points as well as home delivery (compliant with public health rules); (iii) accompaniment and social support (e.g., to offer opportunities for peer-to-peer exchange and learning about technologies). These Centers may also provide faculty members with (i) creative, accessible and user-friendly online participatory tools, methods and platforms and (ii) accompaniment in the provision of appropriate support to online participation. Financial compensation should also be rethought to consider new expenses associated with online participation (e.g., Internet connection fees, document printing).

Finally, since Fricker (2007) outlines the systemic character of epistemic injustice, solutions toward epistemic justice in medical education should also go beyond individual reflexive awareness to challenge the power hierarchy at the *macro* level. Building on the assumptions provided by critical approaches ( Halman, Baker and Ng, 2017), we suggest that decision-makers (i) acknowledge the prevalence and persistence of power issues in medical education, understand their deep roots as well as their consequences on students, professionals, educators and patients and (ii) engage by questioning the status quo and establishing a multilevel agenda for action (that extends across medical education policy, management and practice) to encourage epistemic justice in medical education. This process should involve authentic dialogue with patients from diverse contexts and social backgrounds, the recognition of the value and authority of these patients’ contribution, in order to co-design, co-develop and co-implement a renewed and innovative curriculum, sustainably involving patients as epistemic agents.

## Conclusion

Based on Fricker’s (2007) perspective on epistemic injustices, this article aimed to highlight issues associated with the epistemic exclusion of patients’ knowledge in medical education in the context of COVID-19 and to suggest promising strategies to address these issues going forward. Patient engagement in medical education is essential to bridge the gap between general biomedical knowledge and patients’ singular experiential knowledge. Strengthening this educational practice and ensuring its sustainability are key to fostering desirable outcomes such as a more humane, compassionate and just medical practice, based on the acknowledgement of the value of different but complementary types of knowledge and experiences.

## Take Home Messages

• Epistemic injustices are power inequalities in the access, recognition and production of knowledge. In medical education, they traditionally translated into power imbalances that excluded and alienated patients, and their experiential knowledge.
• Patient engagement is a desirable strategy to address epistemic injustices in medical education.
• Patient exclusion in times of crisis highlights pervasive power imbalances, which needs to be recognized and addressed to reach sustainable epistemic justice in medical education.
• Strengthening patient engagement in medical education and ensuring its sustainability are key to fostering a more humane, compassionate and just medical practice, based on the valorization of different knowledge and experiences.

## Notes On Contributors

**Julie Massé** is a doctoral candidate in Community Health, Faculty of Nursing, and student researcher with the Office of Education and Continuing Professional Development, Faculty of Medicine, Université Laval, Québec, Quebec, Canada. She is also a student member of *VITAM, Centre de recherché en santé durable,* research center affiliated withUniversité Laval *.*

**Guy Poulin** is a patient-partner involved in several projects and committees in the health sector (including in medical education). In particular, he is involved in various projects of *VITAM, Centre de recherché en santé durable,* research center with which JM and MCT are affiliated.

**Marilyne Côté** is also a patient-partner involved in several research and intervention projects (in medical education, community health and social medicine, among others). In particular, she is involved as a “life experience expert” within the *Mobilisation régionale agir ensemble contre les préjugés.*

**Marie-Claude Tremblay** is Assistant Professor, Department of Family Medicine and Emergency Medicine, and researcher with the Office of Education and Continuing Professional Development, Faculty of Medicine, Université Laval, Québec, Quebec, Canada. She is also a regular member of *VITAM, Centre de recherche en santé durable,* research center affiliated withUniversité Laval.
