# Patient Engagement in Medical Education During the COVID-19 Pandemic: A Critical Reflection on an Epistemic Challenge

**DOI:** 10.15694/mep.2021.000103.2

**Published:** 2021-09-30

**Authors:** Julie Massé, Guy Poulin, Marilyne Côté, Marie-Claude Tremblay

**Affiliations:** 1 Université Laval; 2 VITAM; 3 VITAM; 4 Mobilisation régionale agir ensemble contre les préjugés

**Keywords:** Epistemic Injustice, Patient Engagement, Patient Involvement, Patient Participation, Experiential Knowledge, Medical Education, Power, Reflexivity, Online Teaching, COVID-19

## Abstract

This article was migrated. The article was marked as recommended.

Epistemic injustices are defined as power inequalities in the access, recognition and production of knowledge. Their persistence in medical education, especially to the detriment of patients and their specific knowledge, has been documented by several authors. Patient engagement is a new paradigm that involves fostering meaningful patient collaboration at different levels of the healthcare system. Since it is fundamentally based on the recognition of the value and relevance of patients’ experiential knowledge, patient engagement in medical education is generally recognized as a desirable strategy to address epistemic injustices in the field. Patient engagement is challenged in the context of COVID-19 where most Canadian medical schools have had to quickly modify their teaching models, stop in-person classes and redirect most activities online. This article presents a critical reflection on the issues raised by COVID-constrained teaching strategies and their impact on epistemic injustices in medical education. It also suggests strategies to favour epistemic justice in medical education despite the pandemic turmoil and online shift. It therefore adds an epistemic perspective to the reflection on the effects of the pandemic on medical education and training, which has been little discussed so far.

## Background

The notion of epistemic injustice has emerged under contemporary feminist and postcolonial epistemologies to help rethink the links between social inequalities and knowledge production ( Godrie and Dos Santos, 2017). On this basis, epistemic injustices were defined as inequalities of power in the access, recognition and production of knowledge ( Fricker, 2007; Godrie and Dos Santos, 2017). These injustices fundamentally arise from broader systemic inequalities and situations of oppression structuring and nurturing social relations in various contexts ( Godrie and Dos Santos, 2017). Their persistence in medical education, especially to the detriment of patients and their specific knowledge, has been documented by several authors ( Bleakley, Bligh and Browne, 2011; Sharma, 2018). It has namely been argued that traditional social dynamics in medical education, rooted in the long-lasting culture of a health system that has fundamentally built itself on expert knowledge ( Dumez and Pomey, 2019), “led to and perpetuated the types of power imbalances that excluded and alienated patients” ( Bleakley, Bligh and Browne, 2011, p. xiii).

Patient engagement is a new paradigm that involves fostering meaningful patient collaboration at different levels of the healthcare system, including direct care, health service organization, health policies and medical education ( Pomey *et al.*, 2015). It is the culmination of a logical evolution, over several decades, of ways of thinking about health and organizing health care and services ( Dumez and Pomey, 2019). Since it is fundamentally based on the recognition of the value and relevance of patients’ experiential knowledge ( Tuckett *et al.*, 1985; Pomey *et al.*, 2015; Dumez and Pomey, 2019), patient engagement in medical education is generally recognized as a desirable strategy to address epistemic injustices in the field. Indeed, meaningful patient participation (i.e., going beyond the historically instrumental role granted to patients in medical education) can enable patients to bring needed experiential knowledge in the training of future physicians and to consequently constitute themselves as holders and creators of valued knowledge.

That said, patient engagement is challenged in the context of COVID-19 where most Canadian medical schools have had to quickly modify their teaching models, stopping in-person classes and redirecting most activities online to allow social distancing and curb the spread of the virus. In Quebec (Eastern Canadian province), the Premier spoke to the citizens on March 12, 2020, to announce a first wave of public health measures. The following day, the provincial Ministry of Education and Higher Education confirmed the suspension of all classroom training and evaluation activities. He also confirmed the introduction of specific measures applicable to the province’s universities with respect to the maintenance of distance learning. Although government guidelines have evolved over time and by regions since then, training activities in Quebec universities remain mostly online at the time of writing this article. The situation is comparable in other Canadian provinces. Some relaxations are expected in the fall of 2021.

In this article, we present a critical reflection on the issues raised by COVID-constrained teaching strategies and their impact on epistemic injustices in medical education. We also suggest strategies to favour epistemic justice in medical education despite the pandemic turmoil and online shift. The article therefore adds an epistemic perspective to the reflection on the effects of the pandemic on medical education and training, which has been little discussed so far.

This reflection finds its importance in the role that patient engagement plays in the promotion of fundamental learning in medical learners. For instance, studies have reported multiple benefits of patient engagement in medical education, such as changes in students’ attitudes toward patients ( Fitzpatrick *et al.*, 2006; George *et al.*, 2011), new skills, knowledge and understandings related to patient-doctor communication ( Fitzpatrick *et al.*, 2006), as well as new insights of psychosocial aspects of patients’ everyday life with the disease ( Kumagai, Murphy, and Ross, 2009; Shapiro, Tomasa and Koff, 2009). There is also a growing body of scientific evidence identifying patient engagement as a way to promote reflective learning in medical learners by questioning social positions and standards taken for granted, and promoting awareness of patient diversity in profiles, health trajectories and needs ( Towle *et al.*, 2010). As such, patient engagement in medical education can promote transformative healthcare practices.

## Fricker’s theoretical proposition on epistemic injustices

Our critical reflection will be based on the theoretical proposition of Miranda Fricker (2007), a contemporary British philosopher whose work is at the very origin of the notion of epistemic injustice. According to Fricker (2007), there are two kinds of epistemic injustices: testimonial injustice and hermeneutical injustice. The next sections will attempt to describe these two types of epistemic injustices in further detail and present how they actually apply to patient engagement in medical teaching in the pandemic context.

## Patient exclusion in the pandemic context: source of epistemic injustice?

### Patient exclusion as testimonial injustice

Following Fricker (2007), **testimonial injustice** involves a hearer attributing an unjust credibility deficit to a certain speaker due to prejudice, bias and stereotyping. The author depicts this type of epistemic injustice “as a kind of injustice in which someone is wronged specifically in his/her capacity as a knower” (p. 20). This is, in fact, an illegitimate and prejudicial differential judgement of a person’s testimony based on his/her status. Fricker gives the example of a woman who is sexually harassed, and who is not believed because of a credibility deficit arising from her gender status. Following the same logic, it could also be a patient who shares his/her experience of the disease and who is not taken seriously within the health system because of his/her patient status. Following Fricker (2007), such injustice lies in the fact that some individuals and groups are not considered *good informants* regarding their capacity to produce knowledge that is perceived as valuable from the dominant standpoint. Ultimately, “this operation of identity power controls who can convey knowledge to whom and, by the same token, who can gain knowledge from whom” (p. 90).

### How does testimonial injustice apply to medical education in the COVID-19 context?

One of the first impacts of the COVID-19 crisis in the medical field has been that, in light of the emergency measures that had to be taken, decision-makers redesigned activities in a manner that resulted in the exclusion of patients from many participatory platforms. As outlined by other authors ( Drinkwater, 2020; Richards and Scowcroft, 2020), the way patient participation was quickly and easily stopped in various contexts raises fundamental questions about how it is really valued. From an epistemic perspective, such exclusion highlights pervasive power imbalance in which power holders in the medical field still tend to disqualify patients as epistemic agents, holding valuable knowledge of their own. In the face of adversity, we have indeed quickly returned to traditional often-criticized paternalistic logics ( Bleakley, Bligh and Browne, 2011; Sharma, 2018) where physicians and academics are the sole holders of authority, credibility and legitimacy to create and share knowledge, and where patients are relegated to their status of objects of medical knowledge. In medical education, patient contributions, based on knowledge and skills gained through experiences, has been judged non-essential given the emergency circumstances and therefore excluded from the curriculum in favour solely of biomedical expert knowledge. A whole cohort of medical students is thus deprived of privileged contact with patients and the fundamental learning that could result from the sharing of their experiential knowledge.

### Testimonial injustices and digital inequalities

In the current pandemic context where most training activities are transferred online, we can also establish a link between testimonial injustices and digital inequalities, whose increase in time of COVID-19 has been demonstrated ( Beaunoyer, Dupéré and Guitton, 2020). In the particular context of medical education, we are aware that some institutions managed to keep patient engagement alive in medical teaching despite the COVID-constrained educational context (for instance, the Université de Montréal Collaboration and Patient Partnership Unit’s initiative in the context of which patient teaching roles have rapidly moved online) ( Descoteaux and Jackson, 2020). One of the most commonly preferred strategy to do so lies in online meeting platforms (e.g., Zoom, Teams, Webex), which now often replace in-class group interactions that took place before the crisis. We clearly see the practicality of such socially-distanced synchronous teaching approaches in the pandemic context. We also see their educational interest facing the rise of the e-patient and the consequent growing need to engage electronically with patients; however, they still raise unavoidable epistemic issues.

Indeed, as digital technologies become the prominent way to interact, participate and learn in medical education under the impetus of the pandemic, inequalities in access to and ability to use technologies may limit the range and scope of patient engagement. Digital inequalities make it potentially complex for people or communities who have difficulty accessing or using technologies due to financial, geographic, social or literacy-related barriers, to access participatory spaces and then properly engage in knowledge co-construction. Therefore, the use of digital participatory tools will be silencing the voice of some patients who will be unjustly denied the opportunity to share their knowledge if they are not given the proper means to do so. Failure to adequately take into account the barriers to online participation faced by some individuals and groups in the design of educational strategies thus represents the basis for testimonial injustices.

Engaging patients experiencing vulnerability in medical education is key to addressing access-to-care inequities. For instance, it may allow students to gain an embodied knowledge and understanding of health issues resulting from social vulnerability, and the social and cultural competencies required to provide acceptable care for all; however, these patients are especially likely to experience barriers to online participation. Engaging them was already a major challenge before the pandemic ( Loignon *et al.*, 2019; Pomey *et al.*, 2019). The shift to online educational activities in medical education is widening disparity since it may reinforce the current trend toward the recruitment of well-resourced, educated and privileged patients ( Sharma, 2018), once again depriving students of fundamental learning.

### Patient exclusion as hermeneutical injustice

Fricker (2007) defines **hermeneutical injustice** as “the injustice of having some significant area of one’s social experience obscured from collective understanding owing to hermeneutical [i.e., interpretative] marginalization” (p. 158). It refers to unjust inequalities in the intelligibility of social experiences, given the collectively-accepted interpretative schemes for understanding the world. Following Fricker (2007), the misinterpretation of one’s social experience often results from his/her traditional exclusion from activities meant to shape collective interpretative schemes. As an example, the author’s work highlights how the experience of some women (e.g., relating to sexual harassment) is less collectively understandable (or intelligible) because women have historically been excluded from activities meant to shape the way we collectively interpret the world (e.g., scholarship, journalism, politics). As Fricker (2007) claims that this is also the case for other marginalized groups, we consider that it is the case of patients within the health systems. Indeed, following the same logic, “[the] expert health system [...] has defined itself for decades without its users, patients, and citizens” ( Dumez and Pomey, 2019, p.14), and patients have thus traditionally been excluded from activities meant to shape the way we collectively understand health and disease.

### How does hermeneutical injustice apply to medical education in the COVID-19 context?

Scientific literature ( Domecq *et al*., 2014; Carroll *et al.*, 2017) suggests that the main challenges to patient engagement often lie in the perceived logical and practical burden associated with it (e.g., the time and energy required to develop a common language, to redefine the roles and to value patient contribution in a way that avoids tokenism). In the face of the pandemic turmoil, we postulate that medical teachers, if they do not exclude patients completely, might be inclined to avoid such a burden and seek alternative strategies to somehow maintain student exposure to patient perspectives in a relatively simple, timely and energy-saving way. For instance, medical teachers may be tempted to fall back on the online broadcasting of patient testimonials. At first glance, the formative character of such practice seems relevant since it contributes to the contextualization of one patient’s case and, in doing so, provides better understanding of the issues raised and fosters the acquisition of the skills required to properly address it ( Lechopier and Granier, 2017); however, it does not restore the patient’s role as co-constructor of knowledge. Indeed, in the absence of meaningful interactive patient-student contacts allowing for authentic dialogue, unilateral patient testimony risks resulting in academic and clinical teachers using the patient’s narrative as a tool to illustrate clinical or biomedical knowledge ( Lechopier and Granier, 2017). In other words, the patient’s narrative acts “as interesting teaching ‘material’, often no more than a medium through which the teacher teaches” ( Spencer *et al*., 2000, p. 851).

In such a context, the hermeneutical injustice lies in the potential inability for medical teachers - and medical students - to make sense of the patient’s experience because the collectively accepted dominant biomedical paradigm they refer to does not fit the paradigm within which the patient’s narrative is forged. At the heart of the medical education curriculum, the biomedical knowledge acquired by future physicians is mainly derived from disciplinary scientific knowledge in the clinical, pathophysiological and epidemiological fields ( Lechopier and Granier, 2017). This knowledge covers diseases, their classification, diagnosis and treatment ( Lechopier and Granier, 2017). In such a paradigm, people’s experience of health will tend to be considered as existing outside the patients-subjects, independently of the knowledge or representations they may have of it. Indeed, the tendency will be to understand health and disease in terms of vital norms and objective indicators, to reduce them to their physiological dimension, in a logic that is disconnected from the patient’s lived and signified reality ( Vonarx and Desgroseilliers, 2013). The patient’s unique experience thus risks being assimilated to an objectified, dehumanized and universalized ‘patient perspective’ ( Rowland *et al.*, 2017; Sharma, 2018), and to be instrumentalized in the reproduction of existing power dynamics and ideological systems rather than possibly being used to resist or transform them ( Rowland *et al.*, 2017).

Practical implications of such hermeneutical injustices relate to the gap that needs to be filled between, on the one hand, general biomedical knowledge that translates into unilateral and universal approaches to care and, and on the other, clinical medical practice, which is aimed at a singular patient who has his/her own conception of health. This highlights the need for renewed educational approaches that foster individualized and sensible healthcare practice.

## Possible solutions toward a sustainable epistemic justice in medical education

We have highlighted how COVID-constrained teaching strategies can exclude patients from the process of knowledge co-construction in medical education and thus constitute a source of epistemic injustice. We have also attempted to highlight the practical implications of such exclusion for medical education. Knowing that COVID-19 will likely affect us for a long time and that many institutions now consider virtual teaching practices as the way forward, the issues raised highlight the need to initiate a reflection on appropriate strategies to develop a sustainable renewed model for active and equitable patient engagement in medical education. Consistent with Fricker’s proposal (2007), we consider that educational strategies should be rooted in the principles of testimonial and hermeneutical justice.

### Developing reflexive awareness and critical thinking

From both testimonial and hermeneutical perspectives, solutions toward epistemic justice, as proposed by Fricker (2007), mainly rely on the hearers’ (i.e., medical teachers and students) development of a reflexive awareness to ultimately change how he or she perceives and engages with the speaker (i.e., the patient).

From the testimonial perspective, the reduction of epistemic inequalities in medical education may be achieved through solutions “neutraliz[ing] the impact of all sorts of prejudices endemic in the climate of testimonial exchange” ( Fricker, 2007, p. 96). Following Fricker (2007), such neutralization would require medical students and teachers to engage in an active critical reflection to question their spontaneous perception of the patient’s words, to become aware that this perception is not neutral since it is the result of an identity-based power that mediates between them and the patient and to strategize to “compensate upwards” (p. 91-92).

As for solutions based on hermeneutical justice, they will seek to promote the hearers’ development of a reflexive awareness for the existence of gaps in collective interpretative resources that can be mobilized to understand some speakers’ message ( Fricker, 2007). Indeed, medical teachers and students may reflexively question how their social identity, the very foundations of their interpretative schemes, the biomedical paradigm they were socialized in and the power dynamics it involves, limit their in-depth understanding of the patient’s discourse and potentially make them miss out on fundamental learning.

### Addressing digital inequalities

Following Fricker (2007), in order to support the hearers’ reflexive efforts, educational strategies promoting “a more inclusive hermeneutical micro-climate through the appropriate kind of dialogue” (p. 171) between teachers, students and patients should be prioritized within the medical curriculum. This implies that interventions involving “a more pro-active and more socially aware kind of listening than is usually required in more straightforward communicative exchanges” ( Fricker, 2007, p. 171) should be sustainably implemented and adequately supported. We are in fact talking about creating appropriate social spaces for authentic dialogue between patients, students and teachers. The Université de Montréal’s initiative ( Descoteaux and Jackson, 2020) shows us that the pandemic emergency and the online shift are not necessarily an obstacle to the implementation of such dialogue; however, in that particular context, attention should be paid to digital inequalities to ensure equitable access to knowledge sharing opportunities.

Beaunoyer *et al.*’s (2020) proposition of a set of sustainable action-focused strategies targeting equitable access to and use of technologies may be particularly useful to foster equitable access to online participatory spaces. For instance, Patient Service Centres could be implemented to offer: (i) training courses aimed at increasing patients’ digital literacy; (ii) equipment loan services, with geographically and physically accessible service points as well as home delivery (compliant with public health rules); (iii) accompaniment and social support (e.g., to offer opportunities for peer-to-peer exchange and learning about technologies). These Centers may also provide faculty members with (i) creative, accessible and user-friendly online participatory tools, methods and platforms and (ii) accompaniment in the provision of appropriate support to online participation. Financial compensation should also be rethought to consider new expenses associated with online participation (e.g., Internet connection fees, document printing).

### Challenging the power hierarchy at the macro level

Finally, since Fricker (2007) outlines the systemic character of epistemic injustice, solutions toward epistemic justice in medical education should go beyond individual strategies to promote reflexive awareness and challenge the power hierarchy at the *macro* level. Building on the assumptions provided by critical approaches ( Halman, Baker and Ng, 2017), we suggest that decision-makers (i) acknowledge the prevalence and persistence of power issues in medical education, understand their deep roots as well as their consequences on students, professionals, educators and patients and (ii) engage by questioning the status quo and establishing a multilevel agenda for action (that extends across the medical education policy, management and practice) to encourage epistemic justice in medical education. This process should involve patients from diverse contexts and social backgrounds and the recognition of the value and authority of these patients’ contribution in order to co-design, co-develop and co-implement a renewed and innovative curriculum, sustainably involving patients as epistemic agents.

Along with patients themselves, some patient engagement organisations, such as the Society for Participatory Medicine ( https://participatorymedicine.org) and the Centre of Excellence on Partnership with Patients and the Public ( https://ceppp.ca/en/), might be resourceful partners to support the adoption of the “patient engagement” paradigm in medical schools. Development of partnerships between medical schools and schools of humanities and social sciences could also be a promising avenue. Indeed, humanities and social sciences are often guided by other paradigms and approaches, which would help medical learners and teachers to think differently about patients and their realities.

## Conclusion

Based on Fricker’s (2007) perspective on epistemic injustices, this article aimed to highlight issues associated with the epistemic exclusion of patient knowledge in medical education in the context of COVID-19 and to suggest promising strategies to address these issues going forward. Patient engagement in medical education is a way forward to properly acknowledge the value of different but complementary types of knowledge and experiences, and to address pervasive and detrimental power imbalances in the field. Strengthening this educational practice and ensuring its sustainability are key to fostering desirable outcomes such as a more humane, compassionate and fair medical practice, and to ultimately transform the culture of patient care.

## Take Home Messages

• Epistemic injustices are power inequalities in the access, recognition and production of knowledge. In medical education, they traditionally translated into power imbalances that excluded and alienated patients, and their experiential knowledge.
• Patient engagement is a desirable strategy to address epistemic injustices in medical education.
• Patient exclusion in times of crisis highlights pervasive power imbalances, which needs to be recognized and addressed to reach sustainable epistemic justice in medical education.
• Strengthening patient engagement in medical education and ensuring its sustainability are key to fostering a more humane, compassionate and just medical practice, based on the valorization of different knowledge and experiences.

## Notes On Contributors

**Julie Massé** is a doctoral candidate in Community Health, Faculty of Nursing, and student researcher with the Office of Education and Continuing Professional Development, Faculty of Medicine, Université Laval, Québec, Quebec, Canada. She is also a student member of *VITAM, Centre de recherché en santé durable, *research center affiliated with Université Laval *.*

**Guy Poulin** is a patient-partner involved in several projects and committees in the health sector (including in medical education). In particular, he is involved in various projects of *VITAM, Centre de recherché en santé durable*, research center with which JM and MCT are affiliated.

**Marilyne Côté** is also a patient-partner involved in several research and intervention projects (in medical education, community health and social medicine, among others). In particular, she is involved as a “life experience expert” within the *Mobilisation régionale agir ensemble contre les préjugés.*

**Marie-Claude Tremblay** is Assistant Professor, Department of Family Medicine and Emergency Medicine, and researcher with the Office of Education and Continuing Professional Development, Faculty of Medicine, Université Laval, Québec, Quebec, Canada. She is also a regular member of *VITAM, Centre de recherche en santé durable, *research center affiliated with Université Laval.
