# Supplementary material for: Patient Engagement in Medical Education During the COVID-19 Pandemic: A Critical Reflection on an Epistemic Challenge
Source: MedEdPublish (2016). 2021 Sep 30;10:103. Originally published 2021 Apr 30. [Version 2] doi: 10.15694/mep.2021.000103.2 (PMC10939513; doi:10.15694/mep.2021.000103.2)
Supplement: Peer review history

# Reviewer report: Reviewer response for version 2

**DOI:** 10.21956/mep.20218.r31308

**Published:** 2021-10-08

**Authors:** Richard Hays

**Affiliations:** 1 James Cook University

This review has been migrated. The reviewer awarded 5 stars out of 5 The revised version addresses the comments of reviewers of the previous version. I found this to be an interesting read that perhaps help us understand the 'emptiness' experienced by many during the pandemic. Learners have had restricted access to the wide range of resources - patients, clinicians and observation of/participation in real-world health care. Teachers have been less able to develop helpful relationships with learners and, possibly, patient care may have suffered because of an reduction of challenge and discussion that happens in a learning environment. Technology helps but medical education has become rather two-dimensional and reliant on individual effort. Time will tell if there are any long term impacts of this period. Linking these issues to educational theory is a welcome addition to the literature. I would recommend this article to all involved in teaching and supporting learners.

Reviewer Expertise:

NA

No decision status is available

# Reviewer report: Reviewer response for version 2

**DOI:** 10.21956/mep.20218.r31307

**Published:** 2021-10-05

**Authors:** Ken Masters

**Affiliations:** 1 Sultan Qaboos University

This review has been migrated. The reviewer awarded 5 stars out of 5 The authors have done well to address the reviewers’ most pressing concerns of Version 1, and have produced a strong paper that is engaging, challenging, and yet easier to read. The argument development is clearer and the practical examples provide a firm grounding to the theoretical development.Well done.

Reviewer Expertise:

NA

No decision status is available

# Reviewer report: Reviewer response for version 1

**DOI:** 10.21956/mep.19021.r27306

**Published:** 2021-07-31

**Authors:** Ken Masters

**Affiliations:** 1 Sultan Qaboos University

This review has been migrated. The reviewer awarded 3 stars out of 5 An interesting article on patient engagement in medical education during Covid-19.A difficult topic, indeed, partly because, while medicine frequently sees patients as the direct beneficiaries of medical practice (In Engeström’s model, the “Object”), and so patient engagement is to be expected, it is a little different in medical education. In medical education, the Object is the student, and the Patient is somewhat removed. It is for that reason that students have long been participants in medical education conferences, but patients have had a much smaller presence.Although the paper is grounded strongly in theory (and that is a good thing), I would have liked the authors to take into account a few practical issues, such as:• The rise of the e-patient, since the late 20th century, as a practical contextual factor. This has been building for some time, and the Covid-19 pandemic has accentuated the need to engage electronically with patients, and so there is a need for medical education to address this directly.• Some patient engagement organisations, such as the Society for Participatory Medicine (https://participatorymedicine.org/)• The subtle differences between o educating medical students on the need to include patients in their medical decision-making, and o actually including patients in medical education aspects like curriculum design, teaching, etc.Other issues:• Some of the paragraphs are really long and challenging, especially given the difficulty of the topic, and I would strongly recommend that the authors break up some of their paragraphs, and, perhaps, sign-post them with sub-headings.• Given the complexity (and probably unfamiliarity) of the theories, I would also strongly recommend that the authors try to give more practical examples. This is necessary across the paper, but especially in the first half. (There are some examples given later, but I suspect many readers would be lost before then).Small language error:• “so lied in online”: “lied” should be “lay”Overall, a good paper, but a few things could be implemented in order to make it a little more easily graspable. I look forward to Version 2.

Reviewer Expertise:

NA

No decision status is available

# Reviewer report: Reviewer response for version 1

**DOI:** 10.21956/mep.19021.r27307

**Published:** 2021-07-30

**Authors:** Trevor Gibbs

**Affiliations:** 1 AMEE

This review has been migrated. The reviewer awarded 3 stars out of 5 An interesting, although a difficult paper to read. From an academic point of view, very well researched into an area that places little value on patient involvement. I learned a lot from reading the introduction and found some valuable references to the topic. I am very unsure however that many international readers, whose first language is not English, would find it as easy to read and understand. It gives a very good message based on accepted theory.What I missed completely was the solid, practical implications of these statements. The broader perspective of what should happen next was too broad for me and the suggestions made by the authors did not fulfil my "so what " analysis of these discussions. Academically, a good paper; practically I found it lacking and this is reflected in my star rating

Reviewer Expertise:

NA

No decision status is available
